# Review of the genus *Ceresium* Newman, 1842 (Coleoptera, Cerambycidae) in Fiji

**DOI:** 10.3897/zookeys.532.6070

**Published:** 2015-11-05

**Authors:** Hilda Waqa-Sakiti, Linton Winder, Steven W. Lingafelter

**Affiliations:** 1 Ministry of Fisheries and Forests, Department of Forests, Silviculture & Research Division, Colo-i-suva, Fiji; 2 Department of Forestry and Resource Management, Waiariki Institute of Technology, Rotorua, New Zealand; 3 Systematic Entomology Laboratory, ARS, USDA, National Museum of Natural History, MRC–168, Washington, DC 20560, USA

**Keywords:** Longhorned beetles, endemic species, taxonomy

## Abstract

A taxonomic review of the genus *Ceresium* (Coleoptera: Cerambycidae) found within the Fiji Islands is presented. A total of 17 species is treated. Full morphological descriptions and comparative images of each species are included, along with a dichotomous key for their identification.

## Introduction

Several widespread Cerambycidae genera exist within the Fiji Islands. Among these, the genus *Ceresium* Newman (Cerambycidae: Cerambycinae: Callidiopini) is known to be represented on most oceanic islands by one or two widespread species, with additional local species restricted to either a single island or an island group (Bigger and Schofield 1983). Fiji has the highest number of species in this genus, followed by Papua New Guinea where 13 species are recorded (Bigger and Schofield 1983).

The Fijian *Ceresium* species have received little attention since their initial description. A taxonomic study on the Cerambycidae of the Fiji Islands by Dillon and Dillon (1952) concluded that a more thorough revision of the genus was needed and that the presence of additional species was likely. Lingafelter (2008) concluded, “*Ceresium* is in need of revision, and many species need to be studied more thoroughly as they are known only from their often brief original descriptions.” All of the 17 species treated herein are considered native, with 14 of them endemic to Fiji and three having a much broader distribution.

**Introduction to the Subfamily Cerambycinae; Tribe Callidiopini; Genus *Ceresium*.** The subfamily Cerambycinae has 121 tribes attributed to it globally (Tavakilian and Chevillotte 2015). Beetles within this subfamily can be characterized by the prognathous head, apically expanded palpi, rounded thorax, and relatively slender body. The tribe Callidiopini contains 61 genera globally, of which two genera are recorded for Fiji (*Ceresium* and *Oxymagis* Pascoe). The genus *Ceresium* can be differentiated from *Oxymagis* in the lengths of the antennae with *Ceresium* having its antennae usually as long as its body or longer while *Oxymagis* has antennae almost two thirds the length of its body (Dillon and Dillon 1952). *Ceresium* beetles are usually red-brown to dark brown in color, medium-sized, typically measuring 10–25 mm in total body length, head weakly exserted, and eyes deeply emarginate and pronotum elongate or subquadrate.

The genus *Ceresium* is the most speciose in the tribe, comprising 136 species and subspecies globally (Tavakilian and Chevillotte 2015). The highest diversity of *Ceresium* is found in the southeast Asia region and the Pacific Islands. However, the genus has also been recorded in Africa, Australia, and Papua New Guinea (Gressitt 1959; Hawkeswood 1993), with several additional records from North America and the Caribbean that may represent artificial introductions.

**Biology and ecology of the genus *Ceresium*.** Little has been published on the biology and ecology of *Ceresium*. Duffy (1963), Webb et al. (1988) and Hawkeswood and Dauber (1990) have summarized information available on the biology of the genus. Two species within the genus are widespread species: *Ceresium flavipes* (Fabricius) and *Ceresium unicolor* (Fabricius). *Ceresium unicolor* is widespread in Melanesia being recorded from Waigeo Island, Papua New Guinea, Bismarck Archipelago, Solomon Islands, Vanuatu and Fiji (Bigger and Schofield 1983). Both species are polyphagous in the larval stages, breeding on a range of flowering plants from botanically unrelated families. A study by Hawkeswood and Dauber (1990) indicated that some species are able to adapt well to feeding on the wood and/or sap of foreign plant species as well as native plants.

The habits of adult *Ceresium* are virtually unknown. A study on the species *Ceresium pachymerum* Pascoe in Papua New Guinea by Hawkeswood and Dauber (1990) suggested that adult beetles emerge throughout the year. In addition, adults of *Ceresium pachymerum* appeared to be predominantly nocturnal, often attracted to bright lights around human habitation and usually flying early on warm nights after rain. The adults of *Ceresium pachymerum* do not produce any offensive odours or secretions as do other Cerambycinae, but usually stridulate softly and attempt to bite and arch their antennae repeatedly backwards as a defense mechanism (Hawkeswood and Dauber 1990).

**Distribution of *Ceresium* in Fiji.** Members of the genus in Fiji are known from the islands of Viti Levu, Vanua Levu, Taveuni, Ovalau, Gau, Koro, Kadavu and the Lau group (Dillon and Dillon 1952). Sixteen species have been recorded on the largest island of Viti Levu (Dillon and Dillon 1952; Waqa and Lingafelter 2009). The species *Ceresium gracilipes* Fairmaire is quite widespread throughout most of the Fiji islands and is abundant in the Lau island group. The recently described species *Ceresium tuberculatum* Waqa & Lingafelter, 2009 has been recorded from only two islands; Viti Levu and Gau, being more abundant in Gau Island. Vanua Levu is the second largest island in Fiji yet only *Ceresium gracilipes* has been recorded there. However, this is probably attributable to the lack of insect sampling rather than true absence of species. It is likely that further survey work on the islands of Gau, Koro and Vanua Levu may yield more species with more intensive sampling effort since they have intact forest patches that are likely to harbor representatives of the genus.

## Materials and methods

**Sources and deposition of material.** Holotypes of the species recorded for Fiji and described by Dillon and Dillon (1952) are maintained in the Bernice P. Bishop Museum (BPBM), Hawaii. Those described by Fairmaire (1850; 1881) are deposited in the Muséum national d’Histoire naturelle, Paris, France (MNHN). Additional material examined is deposited in the Natural History Museum, London, UK (NHM), the Smithsonian Institution, Washington DC, USA (USNM) and the Institut royal des Sciences naturelles de Belgique, Brussels, Belgium (IRSB), and the entomological collection at the University of the South Pacific, Fiji (USP).

Where possible, redescriptions were based on examination of holotype specimens. Occasionally, it was necessary to examine photographs of types or original literature as supplemental references—the latter especially if the holotypes or lectotypes could not be found and presumed lost.

**Figure 1.**
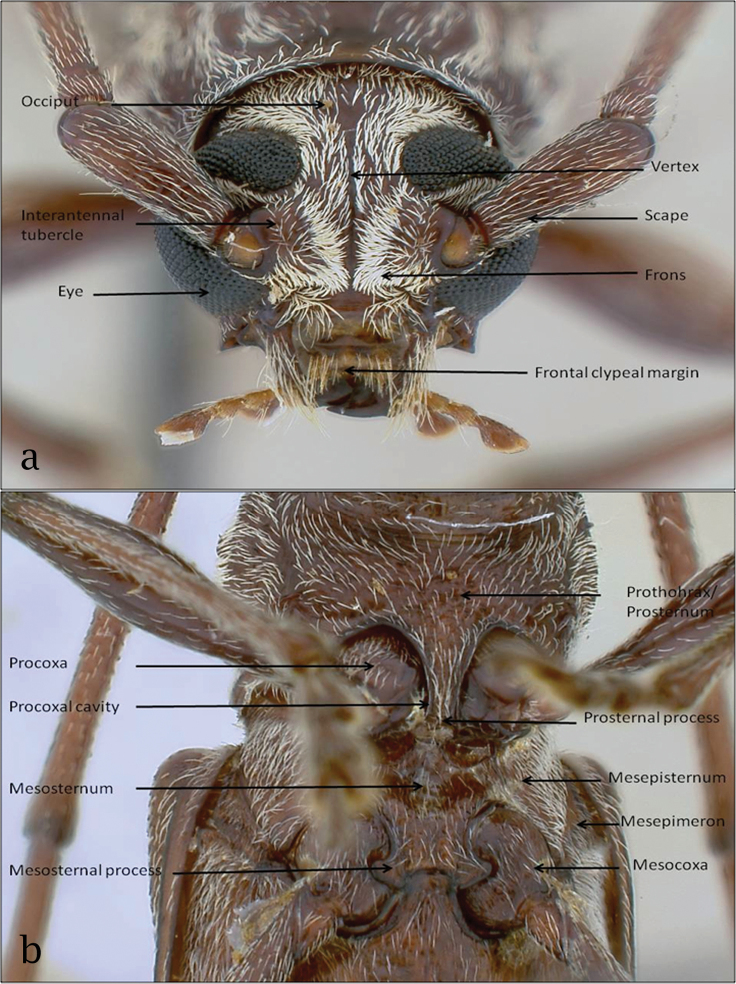
Key anatomy of *Ceresium*: **a** head **b** pro- and mesothorax.

**Species description procedure.** Species descriptions were made using a standard template for each of the 17 species of *Ceresium*. Much of the descriptions of each taxon were updated, but, the original text was retained whenever possible when reviewing each species. Observations include detailed descriptions for the head region (vertex and occiput, antennae, frons and frontoclypeal margin; Figure 1a); pronotal region (elytra, scutellum and legs, Fig. 1b) and the ventral region (venter, prosternal process and mesosternum; Fig. 1b). These morphological characters were observed using a stereomicroscope and any measurements were taken using inbuilt eyepiece graticules. Morphological characters were described consistently across species using a glossary of terms and definitions by Gordh and Headrick (2001).

Original descriptions were also adapted to include detailed high resolution color photographs of each specimen. Photographs of museum voucher specimens were taken using a Leica MZApo stereomicroscope. Extended focus images were taken using a JVC digital camera KY-F70 and Archimed software (Microvision Instruments). Detailed photographs taken for each species included: a) dorsal habitus, b) head region, c) pronotal region and d) ventral region. A key was then developed to provide a means to distinguish individual species.

## Systematics

### Ceresium decorum

Taxon classificationAnimaliaColeopteraCerambycidae

Dillon & Dillon, 1952

Fig. 2

Ceresium decorum : Dillon and Dillon 1952: 22, Fiji: Moala, Vanuka, holotype (BPBM).

#### Redescription.

**Figure 2.**
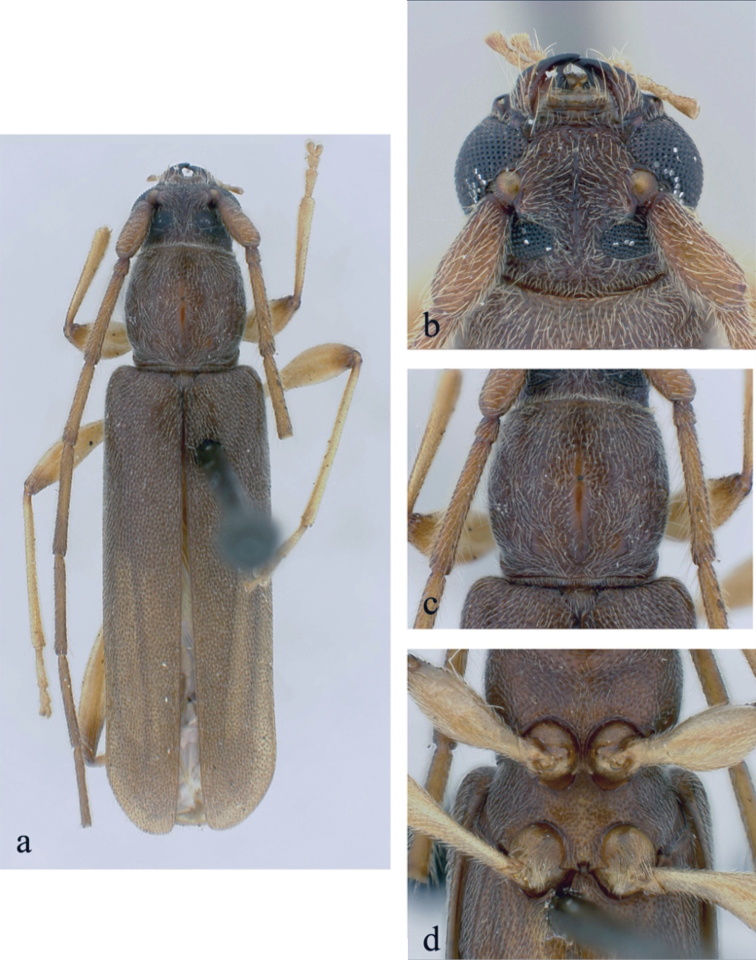
*Ceresium decorum* Dillon & Dillon: **a** dorsal habitus showing integument color (paler towards elytral apex) **b** head detail **c** pronotal detail **d** ventral detail.

Based on the holotype specimen (BPBM) and original description. *Size* 11.7 mm long, 2.8 mm wide at humeri; integument color brown (paler towards elytral apex; Fig. 2a). *Head* with shallow interantennal tubercle region, tubercles only slightly raised; punctate with very sparse ochraceous pubescence on tubercles and throughout frons; vertex and occiput with sparser ochraceous pubescence. Ochraceous pubescence less dense around eye margins. Frons and frontoclypeal margin punctate with sparse, short and long, ochraceous hairs (see head details on Figure 2b). *Antennae* long, extending beyond elytra by three antennomeres. Antennae with vestiture of short, moderately dense, ochraceous setae (longer at apices of antennomeres). Antennomeres unspined and not expanded at apices; Antennomeres 9–11 were damaged. Antennomere 3 and 4 each longer than scape; 5 longest. Very short scape, wide, extending just slightly beyond pronotal front.

*Pronotum* broadly arcuate, widest across middle, and almost as wide as long. Pronotum with glabrous median vertical line, glabrous patch on either basal sides of median line. Pronotum with sparse punctures and sparsely scattered pubescence elsewhere (Fig. 2c). *Elytron* with sparse and regularly spaced ochraceous pubescence. Punctation dense, shallow and gradually becoming shallower and smaller in size towards apex. Elytral apex rounded to suture. Scutellum triangular, narrowly rounded, covered with sparse, ochraceous pubescence. *Legs* moderate in length, femora distinctly but gradually clavate, length of hind femora (3.64 mm), base of femur extending to apical margin of 5^th^ ventrite.

*Venter* of abdomen and thorax with sparse translucent to pale, ochraceous pubescence throughout. Length of abdomen 4.38 mm. Prosternal process very narrow, gradually declivous, weakly notched and expanded at apex, approximately 1/8 width of procoxa. Procoxal cavities widely open posteriorly. Mesocoxae closed laterally to mesepimeron. Mesosternum not produced vertically, without anterior tubercle or sulcus; with weak but acute lateral projections into mesocoxae (Fig. 2d). Apex of terminal ventrite subtruncate without notch.

#### Remarks.

This species is most similar to *Ceresium promissum* Dillon & Dillon based on the key characters. It is distinguished from that species by having the mesosternal process basal notch angled (parallel-sided in *Ceresium promissum*) and the pronotum having a narrow, glabrous, longitudinal line centrally located (restricted to posterior half in *Ceresium promissum*). This species is endemic to Fiji and known only from Moala, Viti Levu, and Lau Islands. It has been collected from rotten logs and dead branches in August and October (Dillon and Dillon 1952).

### Ceresium epilais

Taxon classificationAnimaliaColeopteraCerambycidae

Dillon & Dillon, 1952

Fig. 3

Ceresium epilais : Dillon and Dillon 1952: 23, Fiji: Viti Levu, Colo-i-Suva, holotype (BPBM).

#### Description.

**Figure 3.**
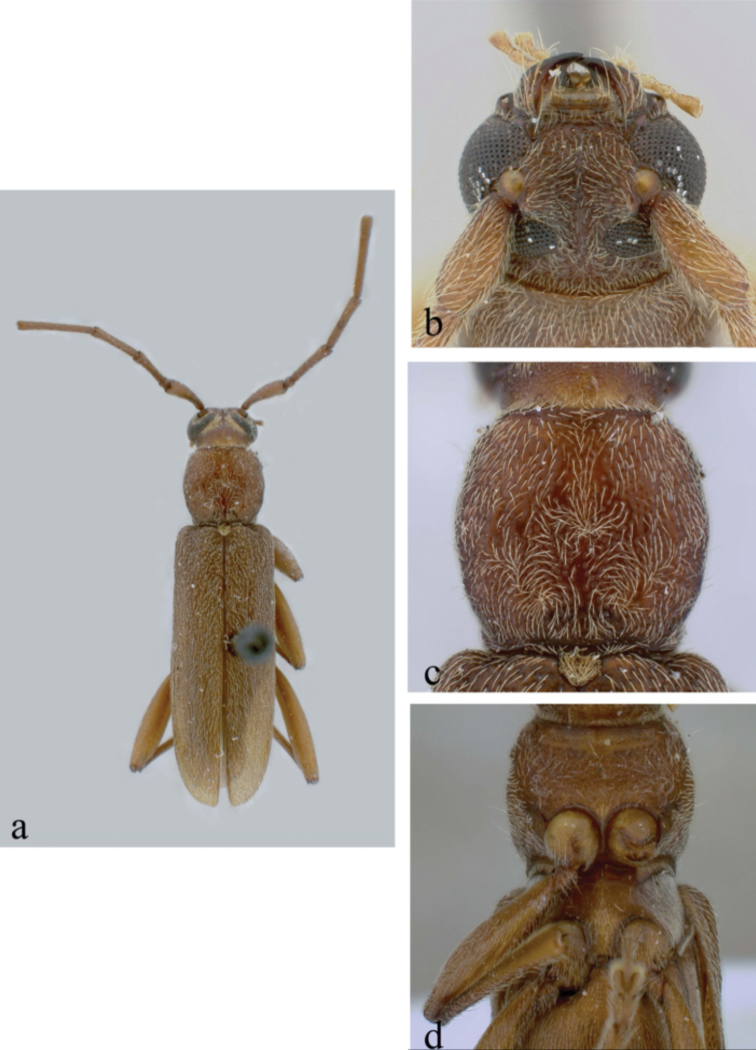
*Ceresium epilais* Dillon & Dillon: **a** dorsal habitus **b** head detail **c** pronotal detail **d** ventral detail.

**Based on the holotype specimen (BPBM) and original description.** *Size* 11.5 mm long, 3.1 mm wide at humeri; integument color brown to light brown (Fig. 3a). *Head* with shallow interantennal tubercle region, tubercles only slightly raised; punctate with very sparse ochraceous pubescence on tubercles and throughout frons; vertex and occiput with sparser ochraceous pubescence. Ochraceous pubescence denser around lower eye margins and around lower antennal insertions. Frons and frontoclypeal margin punctate with sparse, short and long, ochraceous hairs (Fig. 3b). *Antennae* long, extending beyond elytra by two antennomeres. Antennae with vestiture of short, dense, ochraceous setae (longer at apices of antennomeres). Antennomeres unspined and not expanded at apices; Antennomeres 6–11 damaged. Antennomere 3 subequal in length to scape (1.34 mm), Antennomeres 4 and 5 longer than scape, 5 being the longest (2.35 mm). Scape moderate in length (1.34 mm), clavate apically, extending to apical fourth of pronotum.

*Pronotum* broadly arcuate, widest across middle, and slightly wider than long; apex a little narrower than base. Disc densely punctuate with sparsely scattered pubescence elsewhere (Fig. 3c). *Elytron* with subparallel sides, with coarsely dense punctures and regularly spaced ochraceous pubescence. Elytral apex rounded to suture. Scutellum narrowly rounded, covered with dense, rugose (matted), ochraceous pubescence. *Legs* moderate in length (4.02 mm), femora distinctly but gradually clavate, hind femora extending apical margin of 5^th^ ventrite.

*Venter* of abdomen and thorax with mostly sparse, pale ochraceous pubescence throughout, becoming most dense on episternites. Length of abdomen 3.64 mm. Prosternal process very narrow, barely separating and not extending to posterior margin of procoxae; gradually declivous, not expanded at apex, less than 1/15 width of procoxa. Procoxal cavities widely open posteriorly. Mesocoxae closed laterally to mesepimeron. Mesosternum not produced vertically, without anterior tubercle or sulcus; without lateral projections into mesocoxae (Fig. 3d). Apex of terminal ventrite broadly truncate apically without notch.

#### Remarks.

Superficially similar in form and color to *Ceresium vacillans* Dillon & Dillon, it is easily distinguished from that species by having the pronotum more rounded laterally and the elytra lacking glabrous patches. In the key characters, it is most similar to *Ceresium lucidum* Dillon & Dillon, but is distinguished by having the pronotum widest at middle (widest anteriorly in *Ceresium lucidum*). This species is endemic to Fiji and known only from a single specimen collected on Viti Levu in June (Dillon and Dillon 1952).

### Ceresium gracilipes

Taxon classificationAnimaliaColeopteraCerambycidae

Fairmaire, 1881

Fig. 4

Ceresium gracilipes : Fairmaire 1881: 473, Fiji: Ovalau, lectotype (MNHN).

#### Description.

**Figure 4.**
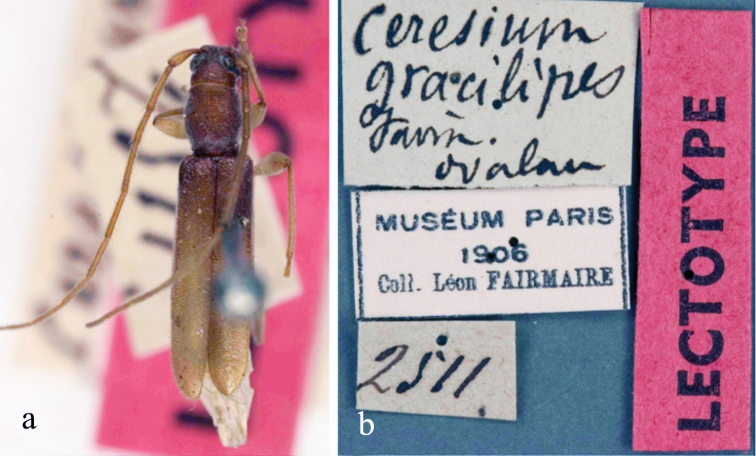
*Ceresium gracilipes* Fairmaire: **a** dorsal habitus **b** lectotype specimen labels.

Based on photograph of lectotype (MNHN) and redescription of Dillon and Dillon (1952). *Size* 4.0–8.5 mm long, 0.7–1.5 mm wide at humeri; elongate-oblong, slender, convex; head and pronotum medium reddish brown, elytra testaceous, laterally darker and with a narrow indistinct fascia behind middle darker; entirely covered with thin pale-fulvous pubescence, which is moderately long; abdomen testaceous (Fig. 4a). Legs testaceous, femora on apical half darker with pubescence as above, antennae slightly darker with slightly longer pubescence. *Head* very coarsely, roughly punctuate. *Antennae* extending beyond half times body length; scape short reaching to apex of pronotum, robust, gradually thickened apically, coarsely punctuate, antennomere 3 slightly shorter than scape, antennomere 4 shorter than 3^rd^; 5^th^ longer than 3^rd^, remaining gradually shorter.

*Pronotum* feebly elongate; base and apex subequal; sides distinctly arcuate, apical sulcus very broad and shallow; an elongate narrow, median callosity at base; disk slightly less coarsely punctuate than head. *Elytra* with sides nearly parallel to apical quarter, apices slightly, narrowly rounded, entire disk coarsely, densely rugose. Scutellum broadly rounded, sparsely pubescent with an indistinct, dark macula postmedially. *Legs* moderate in length, femora moderately clavate with hind femora extending well before elytral apex.

*Venter* with pubescence unexamined. *Prosternum* very narrow between procoxae and broadly expanded apically. *Mesosternum* process wide and strongly expanded at apex, apical margin subtruncate, angle fitting into grooves in mesocoxae; mesocoxae subcontiguous. Fifth sternite attenuate, apical margin strongly emarginate in male; subtruncate in female.

#### Remarks.

This species is recognized by the relatively narrow, parallel-sided appearance and elytra with an indistinct, postmedial dark macula that extends toward the base. It is distinguished from the similar *Ceresium olidum* (Fairmaire) by having the elytra more finely punctate and the pronotum less regularly and densely punctate. This species is endemic to Fiji and known from Viti Levu, Taveuni, Ovalau, and the Lau Islands where it has been collected by beating shrubs from August through November (Dillon and Dillon 1952).

### Ceresium grandipenne

Taxon classificationAnimaliaColeopteraCerambycidae

Fairmaire, 1881

Fig. 5a–d

Ceresium grandipenne : Fairmaire 1881: 472, Fiji: Viti Levu, holotype (MNHN).

#### Description.

**Figure 5.**
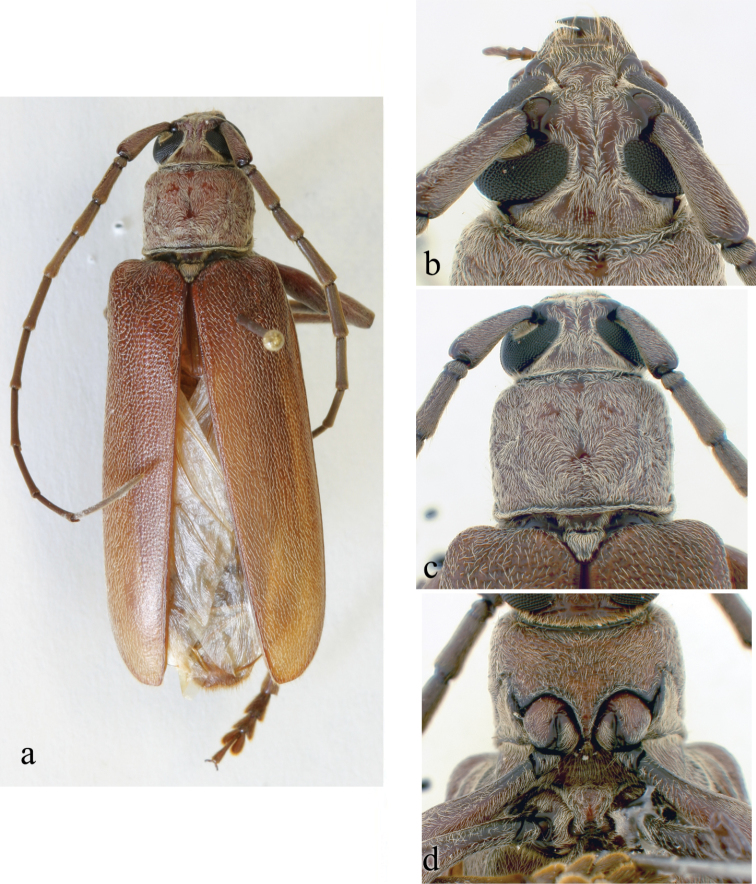
*Ceresium grandipenne* Fairmaire: **a** dorsal habitus **b** head detail **c** pronotal detail **d** ventral detail.

Based on a specimen housed in USP matching original description (the holotype at MNHN is lost). *Size* 21.5–34.0 mm long, 6.0–8.5 mm wide at humeri; integument light brown to brown (Fig. 5a). *Head* with shallow interantennal tubercle region, tubercles only slightly raised; punctate with very sparse ochraceous pubescence on tubercles and throughout frons except center being glabrous; vertex and occiput with moderately dense ochraceous pubescence. Ochraceous pubescence denser around eye margins. Frons and frontoclypeal margin moderately dense, coarsely punctate with sparse, long, ochraceous hairs (Fig. 5b). *Antennae* long, just almost reaching elytral apex. Antennae with vestiture of short, dense, ochraceous setae (longer at apices of antennomeres). Antennomeres unspined and expanded at apices; last antennomere about 1.6 times length of penultimate. Antennomere 3–10 each shorter than scape, 3 being the shortest; 11 longest. Scape long, clavate, extending to apical eighth of pronotum.

*Pronotum* quadrate, slightly widest at apical third, and wider than long; not tuberculate; calli absent. Pronotum with moderately dense ochraceous pubescence throughout and moderately dense punctures (Fig. 5c). *Elytron* with sparse, evenly spaced ochraceous pubescence throughout. Punctation shallow, moderately dense gradually becoming shallower and indistinct towards apex. Elytral apex rounded to suture. Scutellum triangular, covered with dense, ochraceous pubescence. *Legs* moderate in length, femora distinctly but gradually clavate, hind femora extending to base of third ventrite.

*Venter* of abdomen and thorax with moderately dense, ochraceous pubescence at sides, sparse ochraceous pubescence along middle; prosternum sparsely pubescent throughout and on sides. Prosternal process narrow, vertical and acutely declivous, about 1/6 width of procoxa, strongly notched and expanded at apex. Procoxal cavities open posteriorly. Mesocoxae closed laterally to mesepimeron. Mesosternum rather acutely declivous, with small anterior tubercle, and sulcate anteriorly. Mesosternal apex expanded circularly and inserted into mesocoxa (Fig. 5d). Metasternum with a distinct median line running longitudinally along middle. Apex of terminal ventrite truncate to unevenly rounded, without notch.

#### Remarks.

There is some inconsistency between the original description (Fairmaire 1881) and redescription (Dillon and Dillon 1952) of this rarely collected species. The latter states that the pronotum is much narrower anteriorly than at base and possesses a tubercle on each side, however our examination of a specimen matching the original description reveals the pronotum to be quadrate, without lateral tubercles. The large size of this species, along with the quadrate pronotum and opaque integument are distinctive. This species is endemic to Fiji and known only from Viti Levu. Specimens have been collected from August through October, mostly at lights (Dillon and Dillon 1952).

### Ceresium guttaticolle

Taxon classificationAnimaliaColeopteraCerambycidae

(Fairmaire, 1850) rev. stat.

Fig. 6

Hesperophanes guttaticollis : Fairmaire 1850: 63, Tahiti, holotype (MNHN).
Ceresium guttaticolle yapense : Gressitt 1956: 86, Micronesia: Yap Islands, holotype (USNM).

#### Description.

**Figure 6.**
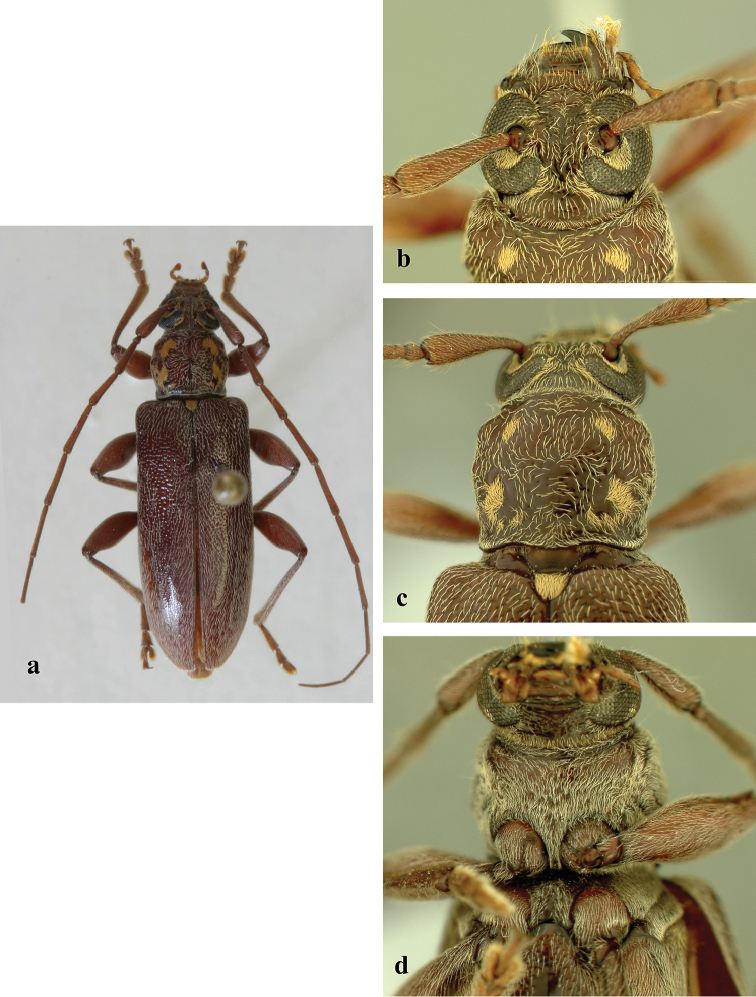
*Ceresium guttaticolle* (Fairmaire): **a** dorsal habitus **b** head detail **c** pronotal detail **d** ventral detail.

Based on the holotype (MNHN), holotype of the subspecies *yapense* Gressitt (USNM), and four specimens from 1988 and 2008 surveys (FNIC, USP). *Size* 12.5–15.0 mm long, 3.0–3.5 mm wide at humeri; integument color orangish-brown (occasionally maroon-brown) (Fig. 6a). *Head* with shallow interantennal tubercle region, tubercles only slightly raised; punctate with very sparse ochraceous pubescence on tubercles and throughout frons; vertex and occiput with sparser ochraceous pubescence. Ochraceous pubescence denser around eye margins. Frons and frontoclypeal margin punctate with sparse, short and long, ochraceous hairs (Fig. 6b). *Antennae* long, extending beyond elytra by 1 antennomere. Antennae with vestiture of short, dense, ochraceous setae (longer at apices of antennomeres). Antennomeres unspined and not expanded at apices; last antennomere approximately 1.3 times length of penultimate. Antennomere 3 and 4 each shorter than scape; 5 and 6 longest except for 11 and subequal in length. Scape long, clavate, extending to apical fifth of pronotum.

*Pronotum* broadly arcuate, widest across middle and slightly wider than long; small tubercles at sides located at middle of sides and anterolaterally. Pronotum with two dense yellow patches of pubescence on either side of pronotum almost subequal in size. Pronotum with sparse punctures and sparsely scattered pubescence elsewhere (Fig. 6c). *Elytron* with sparse and regularly spaced ochraceous pubescence. Punctation dense, shallow and gradually becoming shallower and smaller in size towards apex. Elytral apex rounded to suture. Scutellum narrowly rounded, covered with dense, yellow pubescence. *Legs* moderate in length, femora distinctly but gradually clavate, hind femora extending to between 4^th^–5^th^ ventrite.

*Venter* of abdomen and thorax with moderately dense, ochraceous pubescence throughout becoming less abundant towards 5^th^ ventrite. Prosternal process broad, vertical and acutely declivous, approximately 1/5 width of procoxa, weakly notched and not expanded at apex. Procoxal cavities open posteriorly. Mesocoxae closed laterally to mesepimeron (Fig. 6d). Mesosternum rather acutely declivous, with small anterior tubercle, and sulcate anteriorly. Apex of terminal ventrite subtruncate without notch.

#### Remarks.

Although first described under the name *Hesperophanes guttaticollis*, the holotype actually has a label indicating “*guttatus*”. This is one of the easiest species to recognize due to the yellow pubescent maculations on the pronotum. Only one other species, *Ceresium nigroapicale* Dillon & Dillon has this feature. *Ceresium guttaticolle* has two patches on either side, subequal in length, while *Ceresium nigroapicale* has three or four areas of yellowish pubescence on either side, with the apical noticeably larger. This species was originally described from Tahiti and is also known from Viti Levu, Taveuni, and the Lau Islands in Fiji. The subspecies *Ceresium guttaticolle yapense* Gressitt, 1956 is known from the Yap Islands, Micronesia. Specimens have been collected, mostly at lights, from July through October (Dillon and Dillon 1952). We remove this from synonymy with *Ceresium unicolor* (Fabricius, 1787).

### Ceresium lucidum

Taxon classificationAnimaliaColeopteraCerambycidae

Dillon & Dillon, 1952

Fig. 7

Ceresium lucidum : Dillon and Dillon 1952: 25, Fiji: Viti Levu, holotype (BPBM).

#### Description.

**Figure 7.**
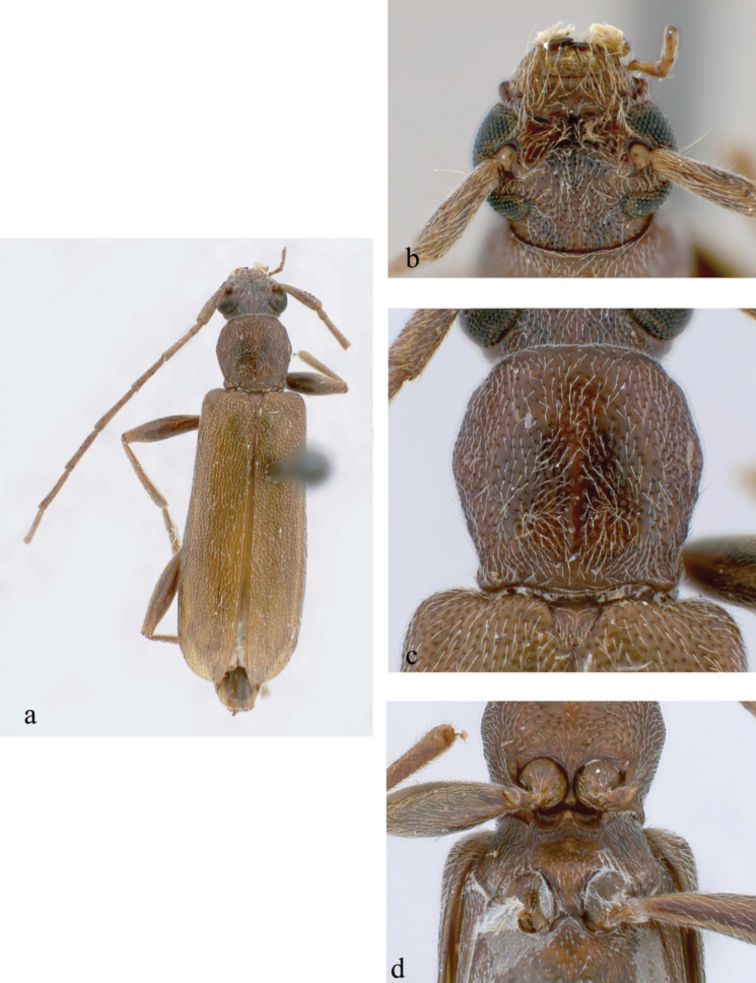
*Ceresium lucidum* Dillon & Dillon: **a** dorsal habitus **b** head detail **c** pronotal detail **d** ventral detail.

Based on the holotype specimen (BPBM). *Size* 9.5 mm long, 2.0 mm wide at humeri; integument color reddish brown, lighter brown towards elytral apex (Fig. 7a). *Head* with shallow interantennal tubercle region, tubercles only slightly raised; punctate with sparse ochraceous pubescence on tubercles and throughout frons; vertex and occiput with sparse ochraceous pubescence. Frons and frontoclypeal margin punctate with sparse and long, ochraceous hairs (Fig. 7b). *Antennae* long, extending beyond elytra by < 2 antennomeres. Antennae with vestiture of short, dense, ochraceous setae. Antennomeres unspined and not expanded at apices; antennomeres 9–11 damaged. Antennomere 4 almost subequal to scape (0.9 mm); 5 longest. Scape very short, gradually clavate, extending slightly beyond apex of pronotum.

*Pronotum* broadly arcuate, widest across middle, and almost as wide as long. Pronotum with sparse punctures and sparsely scattered pubescence elsewhere (Fig. 7c). *Elytron* with sparse and regularly spaced ochraceous pubescence. Punctation dense, shallow and gradually becoming shallower and smaller in size towards apex. Elytral apex rounded to suture. Scutellum triangular and narrowly rounded covered with sparse, ochraceous pubescence. *Legs* moderate in length, femora distinctly but gradually clavate, base of hind femora (3.02 mm) extending to apical margin of 5^th^ ventrite.

*Venter* of abdomen and thorax sparsely ochraceous pubescent throughout, not obscuring integument. Length of abdomen 3.82 mm. Prosternal process very narrow, gradually declivous, weakly notched and expanded at apex, less than 1/5 width of procoxa. Procoxal cavities widely open posteriorly. Mesocoxae closed laterally to mesepimeron (Fig. 7d). Mesosternum not produced vertically, without anterior tubercle or sulcus; without lateral projections into mesocoxae. Apex of terminal ventrite subtruncate without notch.

#### Remarks.

The key characters show this species to be closest to *Ceresium epilais*. *Ceresium lucidum* is distinguished by having the pronotum with a diffuse, dark macula at either side of middle, and being widest anterior of the midpoint while *Ceresium epilais* lacks pronotal maculae and is widest medially at sides. This rare species is endemic to Fiji and known only from the holotype that was collected on Viti Levu at lights during October (Dillon and Dillon 1952).

### Ceresium nigroapicale

Taxon classificationAnimaliaColeopteraCerambycidae

Dillon & Dillon, 1952

Fig. 8

Ceresium nigroapicale : Dillon and Dillon 1952: 27, Fiji: Viti Levu, holotype (BPBM).

#### Description.

**Figure 8.**
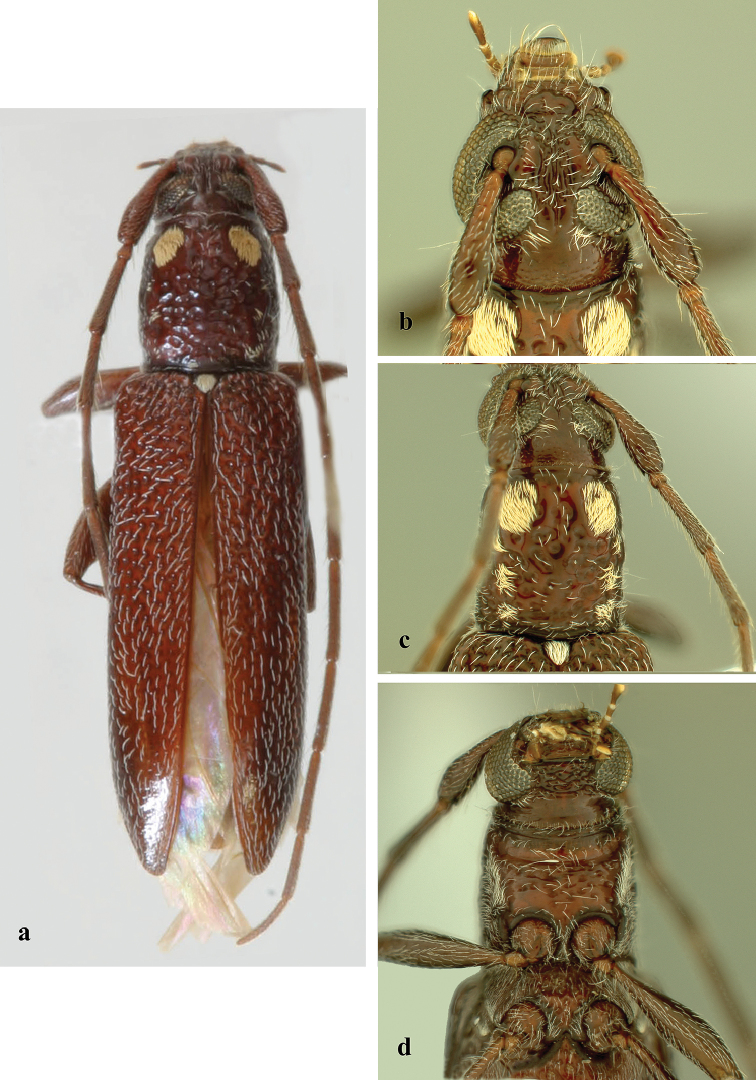
*Ceresium nigroapicale* Dillon & Dillon: **a** dorsal habitus (holotype, BPBM) **b** head detail **c** pronotal detail **d** ventral detail.

Based on the holotype specimen (BPBM) and two specimens from 2008 surveys (FNIC, USP). *Size* 8.5–12.0 mm long, 2.0–2.5 mm wide at humeri; integument color maroonish-brown (Fig. 8a). *Head* with shallow interantennal tubercle region, tubercles only slightly raised; punctate with very sparse ochraceous pubescence on tubercles and throughout frons; vertex and occiput bare. Ochraceous pubescence sparsely around eye margins. Frons and frontoclypeal margin coarsely punctate with sparse, short and long, ochraceous hairs (Fig. 8b). *Antennae* long, extending beyond elytra by almost two antennomeres. Antennae with vestiture of short, dense, ochraceous setae (longer at apices of antennomeres). Antennomeres unspined and not expanded at apices; last antennomere almost subequal in length of penultimate. Antennomere 3 and 4 each shorter than scape; 5–9 longest except for 10–11 and subequal in length. Scape long, clavate, extending to apical fifth of pronotum.

*Pronotum* cylindrical, widest at basal third and slightly longer than wide. Pronotum with four dense patches of yellow pubescence on either side. Apical one largest in size and remaining three almost subequal in size, all arranged longitudinally on either side of pronotum. Pronotum glabrous at center with a few scattered yellow setae with large irregular punctures (Fig. 8c). *Elytron* with sparse, evenly spaced ochraceous pubescence. Punctation shallow, sparse, gradually becoming shallower and indistinct towards apex. Elytral apex rounded to suture. Scutellum narrowly rounded, covered with dense, ochraceous pubescence. *Legs* small to moderate in length, femora distinctly but gradually clavate, hind femora extending to base of fourth ventrite.

*Venter* of abdomen and thorax with sparse ochraceous pubescence throughout. Prosternal process narrow, approximately 1/4 width of procoxa, notched and expanded at apex. Procoxal cavities open posteriorly (Fig. 8d). Mesocoxae closed laterally to mesepimeron. Mesosternum slightly declivous, without anterior tubercle, and sulcate anteriorly. Apex of terminal ventrite truncate to unevenly rounded, without notch. In males, fifth sternite narrow elongate, with a deep V-shape emargination medially extending basally more than one-half its length.

#### Remarks.

This is one of two species characterized by dense pubescent maculae on the pronotum, the other being *Ceresium guttaticolle*. That species has two patches on either side, subequal in length, while *Ceresium nigroapicale* has three or four areas of yellowish pubescence on either side, with the apical noticeably larger. *Ceresium nigroapicale* is further distinguished by the elytra having diffusely darker coloration apically and laterally and very coarse punctation on the basal half. This is another rare species that is endemic to Fiji and known only from Viti Levu. Specimens have been collected in July and September by beating vegetation (Dillon and Dillon 1952).

### Ceresium olidum

Taxon classificationAnimaliaColeopteraCerambycidae

(Fairmaire, 1850)

Fig. 9

Hesperophanes olidus : Fairmaire 1850: 63, Tahiti, holotype (MNHN).

#### Description.

**Figure 9.**
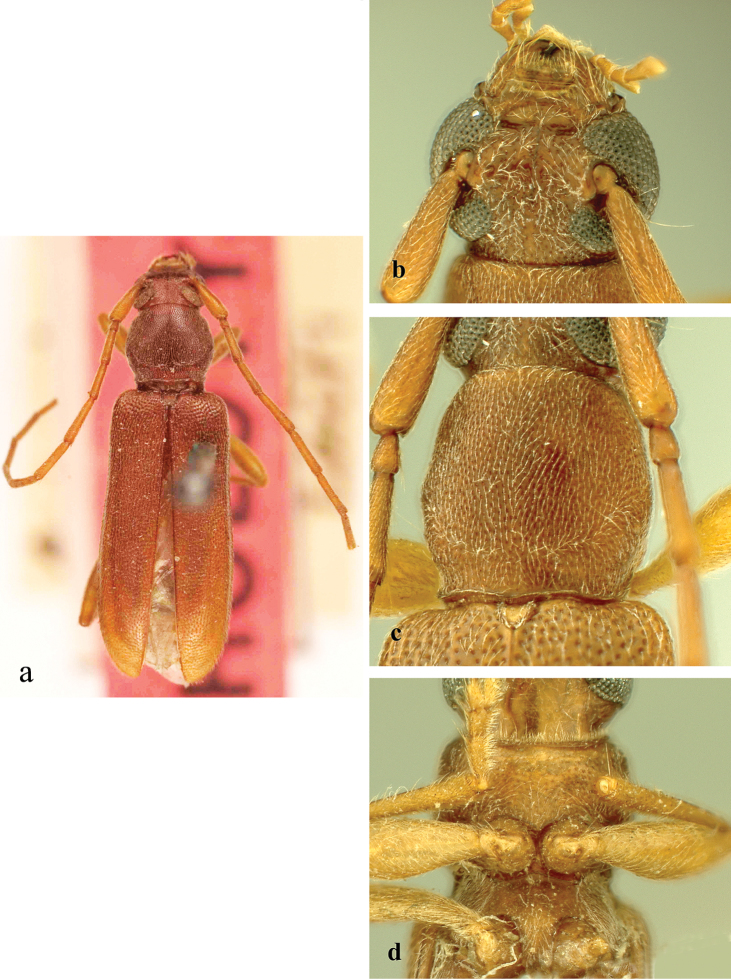
*Ceresium olidum* (Fairmaire): **a** dorsal habitus (holotype, MNHN) **b** head detail **c** pronotal detail **d** ventral detail.

Based on a photograph of the holotype specimen (MNHN) and two specimens from 2008 surveys (FNIC, USP). *Size* 6.5–7.0 mm long, 1.0–1.5 mm wide at humeri; integument color orangish-brown (Fig. 9a). *Head* with shallow interantennal tubercle region, tubercles only slightly raised; punctate with very sparse ochraceous pubescence on tubercles and throughout frons; vertex and occiput with sparser ochraceous pubescence. Frons and frontoclypeal margin punctate with sparse, long, ochraceous hairs (Fig. 9b). *Antennae* long, extending beyond elytra by two antennomeres. Antennae with vestiture of short, dense, ochraceous setae. Antennomeres unspined and slightly expanded at apices; last antennomere almost subequal the length of penultimate. Antennomere 3 and 4 each shorter than scape; 5 longest. Scape long, clavate, extending to apical fifth of pronotum.

*Pronotum* rounded, widest across middle, and as long as wide. Pronotum with fine, sparse and evenly spaced punctures throughout (Fig. 9c). *Elytron* with fine, evenly spaced ochraceous pubescence throughout. Punctation shallow becoming shallower and indistinct towards apex. Elytral apex broadly rounded to suture. Scutellum triangular in shape, covered with sparse, translucent, inconspicuous pubescence. *Legs* small in length, femora distinctly but gradually clavate, hind femora extending to elytral apex.

*Venter* of abdomen and thorax with sparse, ochraceous pubescence throughout. Prosternal process absent. Procoxal cavities open posteriorly. Mesocoxae closed laterally to mesepimeron (Fig. 9d). Mesosternum rather gradually declivous, without anterior tubercle, and sulcate anteriorly. Apex of terminal ventrite truncate to unevenly rounded, without notch.

#### Remarks.

This species is distinguished by having the pronotum with uniform, dense punctation, the third antennal segment extending to about the midpoint of pronotum, and the head, pronotum and scutellum with fine, sparse, ochraceous pubescence. It shares with *Ceresium scutellaris* an incomplete prosternal process between the procoxae. Originally described as *Hesperophanes*, it is known from Viti Levu and the Lau Islands, Fiji and also recorded from Tahiti and Raiatea of the Society Islands of French Polynesia in the original description (Dillon and Dillon 1952; Fairmaire 1850).

### Ceresium promissum

Taxon classificationAnimaliaColeopteraCerambycidae

Dillon & Dillon, 1952

Fig. 10

Ceresium promissum : Dillon and Dillon 1952: 25, Fiji: Viti Levu, Colo-i-Suva, holotype (BPBM).

#### Description.

**Figure 10.**
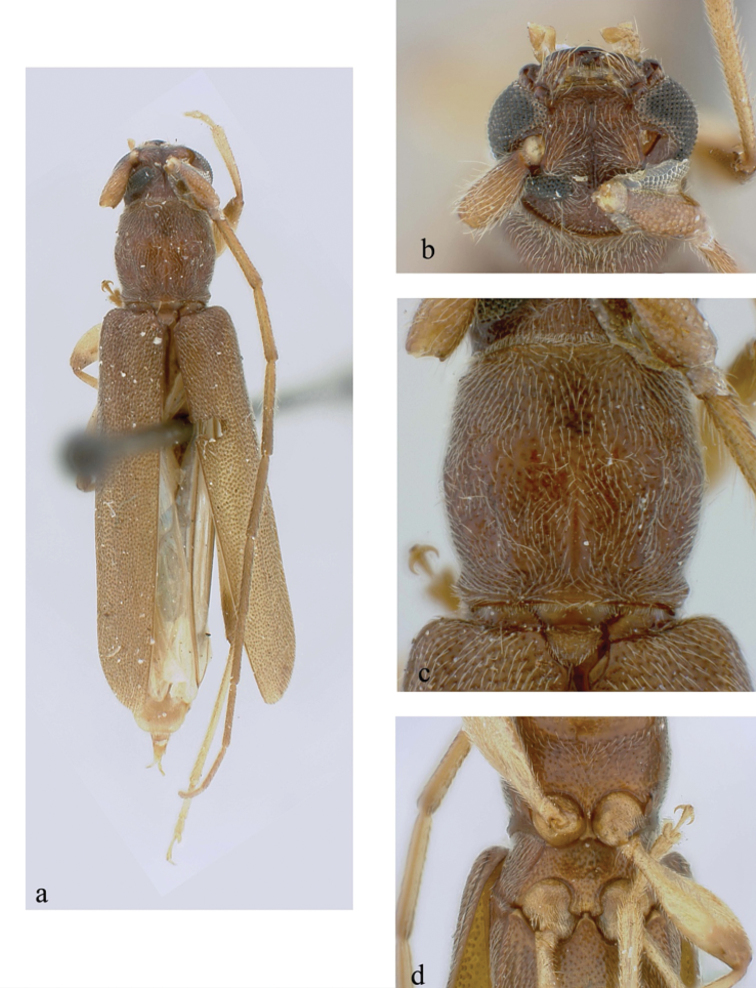
*Ceresium promissum* Dillon & Dillon: **a** dorsal habitus **b** head detail **c** pronotal detail **d** ventral detail.

Based on the holotype specimen (BPBM) and original description. *Size* 8.9 mm long, 1.9 mm wide at humeri; integument color dark brown (pale brown towards elytral apex) (Fig. 10a). *Head* with shallow interantennal tubercle region, tubercles only slightly raised; punctate with very sparse ochraceous pubescence on tubercles and throughout frons; vertex and occiput with sparser ochraceous pubescence. Frons and frontoclypeal margin punctate with sparse, short and long, ochraceous hairs (Fig. 10b). *Antennae* long, extending beyond elytra by two antennomeres. Antennae with vestiture of short, dense, ochraceous setae. Antennomeres unspined and expanded at apices; last antennomere about 1.1 times length of penultimate. Scape shortest in length almost subequal to antennomere 10; 5 longest. Scape short, broad and clavate, extending slightly beyond pronotal front.

*Pronotum* broadly arcuate, widest across middle and slightly longer than wide; glabrous median line, vertically on basal center of pronotum. Pronotum with sparse punctures and sparsely scattered pubescence elsewhere (Fig. 10c). *Elytron* with sparse and regularly spaced ochraceous pubescence. Punctation dense, shallow and gradually becoming shallower and smaller in size towards apex. Elytral apex rounded to suture. Scutellum triangular, narrowly rounded, covered with sparse, short ochraceous pubescence. *Legs* moderate in length, femora distinctly but gradually clavate, hind femora 2.98 mm in length extending to apical margin of 5^th^ ventrite.

*Venter* of abdomen and thorax with sparse, translucent pubescence throughout, not obscuring integument. Length of abdomen 4.04 mm. Prosternal process very narrow, only extending about halfway between procoxae which are nearly contiguous as a result. Procoxal cavities widely open posteriorly (Fig. 10d). Mesocoxae closed laterally to mesepimeron. Mesosternum not produced vertically, without anterior tubercle or sulcus; with very slight lateral projections into mesocoxae. Apex of terminal ventrite subtruncate without notch.

#### Remarks.

By the key characters, *Ceresium promissum* is most similar to *Ceresium decorum*. In *Ceresium promissum*, the mesosternal process has its basal notch parallel-sided and the pronotum has a narrow, glabrous, impunctate line at the middle restricted to the posterior half. In *Ceresium decorum*, the mesosternal process has the basal notch at an angle and the pronotum has the narrow, glabrous, impunctate line at middle centrally located. This species is endemic to Fiji and known only from Viti Levu where the holotype was collected in June (Dillon and Dillon 1952).

### Ceresium pubescens

Taxon classificationAnimaliaColeopteraCerambycidae

Dillon & Dillon, 1952

Fig. 11

Ceresium pubescens : Dillon and Dillon 1952: 19, Fiji: Viti Levu, Tailevu, holotype (BPBM).

#### Description.

**Figure 11.**
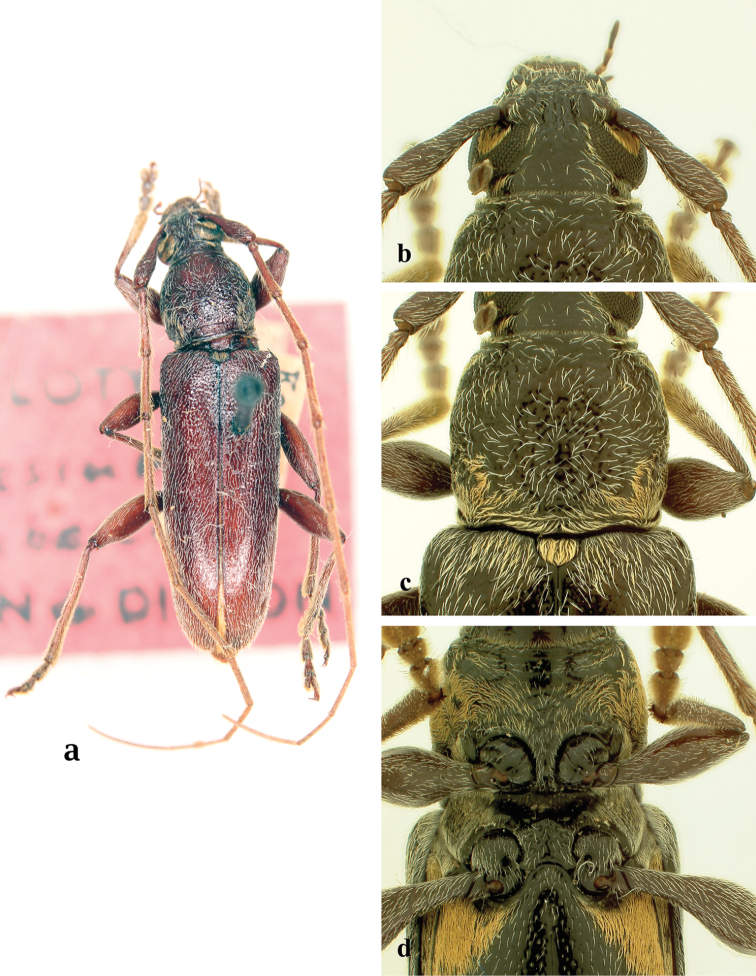
*Ceresium pubescens* Dillon & Dillon: **a** dorsal habitus (holotype, BPBM) **b** head detail **c** pronotal detail **d** ventral detail.

Based on the holotype and a paratype (BPBM) and six specimens from 2005 surveys (FNIC, USP). *Size* 12.0–17.5 mm long, 3.5–4.7 mm wide at humeri; integument color light brown (occasionally piceous) (Fig. 11a). *Head* with shallow interantennal tubercle region, tubercles only slightly raised; punctate with very sparse ochraceous pubescence on tubercles and throughout frons; vertex and occiput with sparser ochraceous pubescence. Ochraceous pubescence slightly denser around eye margins. Frons and frontoclypeal margin densely, coarsely punctate with sparse, short and long, ochraceous hairs (Fig. 11b). *Antennae* long, extending beyond elytra by 1–2 antennomeres. Antennae with vestiture of short, dense, ochraceous setae (longer at apices of antennomeres). Antennomeres unspined and moderately expanded at apices; last antennomere slightly shorter in length to penultimate. Antennomere 3 and 4 each shorter than scape; 5–9 longest and subequal in length. Scape long, clavate, extending to apical fifth of pronotum.

*Pronotum* uniformly arcuate, widest at middle, and slightly wider than long; not tuberculate. Calli absent. Pronotum with sparse yellow pubescence, denser at basal sides; center of disk mostly glabrous. Pronotum with sparse, ill-defined punctures (Fig. 11c). *Elytron* with moderately dense yellow pubescence throughout. Punctation shallow, sparse, gradually becoming shallower and indistinct towards apex. Elytral apex subarcuately rounded to suture. Scutellum broadly rounded, covered with dense, yellow pubescence. *Legs* moderate in length, femora pedunculate clavate, hind femora extending to base of third ventrite.

*Venter* of abdomen and thorax with moderately dense, golden pubescence at sides, but very sparse golden pubescence along middle; except for prosternum which is densely pubescent throughout and on sides. Prosternal process moderately narrow, vertical and acutely declivous, about 1/3 width of procoxa, weakly notched and expanded at apex. Procoxal cavities open posteriorly (Fig. 11d). Mesocoxae closed laterally to mesepimeron. Mesosternum rather acutely declivous, with small anterior tubercle, and sulcate anteriorly. Mesosternum with large punctures. Mesosternal process expanded at apex, distinctly tuberculate and inserted into mesocoxae. Apex of terminal ventrite truncate to unevenly rounded, without notch.

#### Remarks.

One of the characters that define this species is the moderately dense, yellowish pubescence that extends from the sides of the pronotum across the base. In the key, it falls nearest *Ceresium grandipenne*, but it easily distinguished by the much smaller size (always less than 20 mm while *Ceresium grandipenne* is always larger than 21 mm). This species is widespread in Fiji and known from Viti Levu, Ovalau, Moala, and the Lau Islands (Dillon and Dillon 1952).

### Ceresium repandum

Taxon classificationAnimaliaColeopteraCerambycidae

Dillon & Dillon, 1952

Fig. 12

Ceresium repandum Dillon & Dillon, 1952: 16, Fiji: Viti Levu, Nandarivatu, holotype (BPBM).

#### Redescription.

**Figure 12.**
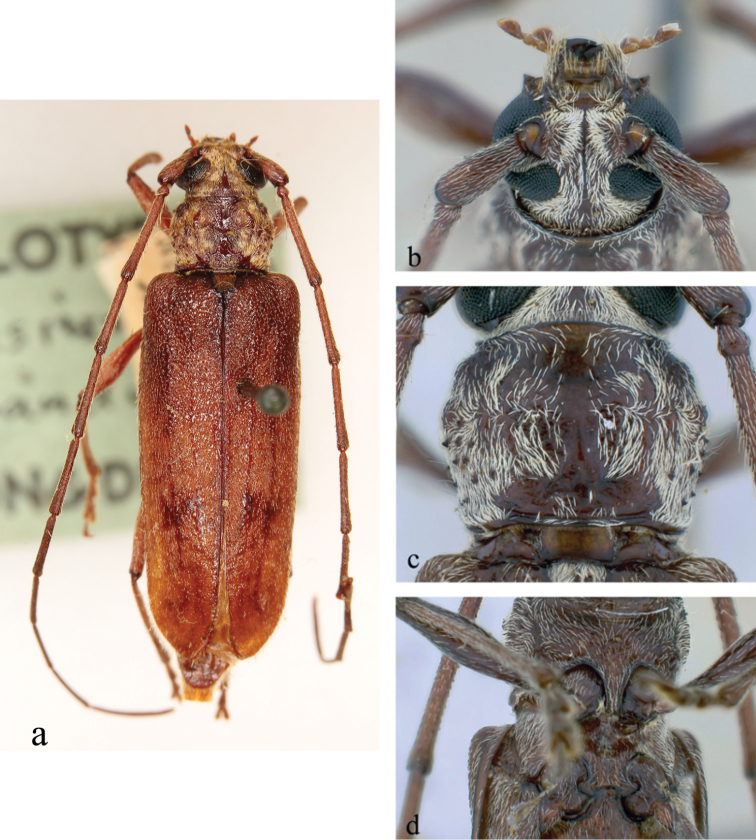
*Ceresium repandum* Dillon & Dillon: **a** dorsal habitus (holotype, BPBM) **b** head detail **c** pronotal detail **d** ventral detail.

Based on the holotype specimen (BPBM) and original description. *Size* 14.3–18.0 mm long, 3.5–5.2 mm wide at humeri; integument color brown to reddish brown (Fig. 12a). *Head* with deep interantennal tubercle region, tubercles raised; punctate with dense ochraceous pubescence on tubercles and throughout frons; vertex and occiput with sparser ochraceous pubescence. Ochraceous pubescence denser around eye margins. Frons and frontoclypeal margin punctate with sparse, short and long, ochraceous hairs (Fig. 12b). *Antennae* long, extending beyond elytral apices by 5 antennomeres. Antennae with vestiture of short, dense, ochraceous setae. Antennomeres unspined and not expanded at apices; last antennomere about 1.3 times length of penultimate. Antennomere 5–6 longest except for 11 (4.12 mm) and subequal in length. Scape short (1.55 mm), clavate, just extending to pronotal apex.

*Pronotum* strongly arcuate, widest across slightly above middle, and slightly wider than long; small tubercles at sides located at the lateral lower half. Pronotum with sparse punctures and dense pubescence laterally (Fig. 12c). *Elytron* with parallel sides and gradually attenuate, with dense and regularly spaced ochraceous pubescence. Punctation sparse, shallow and gradually becoming shallower and smaller in size towards apex. Elytral apex rounded to suture. Scutellum narrowly rounded, covered with dense, ochraceous pubescence. *Legs* moderate in length, femora distinctly but gradually clavate, hind femora (5.02 mm) base extending to apical margin of fifth ventrite.

*Venter* of abdomen and thorax with sparse, ochraceous pubescence throughout, not obscuring integument. Length of abdomen 4.95 mm. Prosternal process narrow, gradually declivous, weakly expanded at apex, approximately 1/4 width of procoxa. Procoxal cavities open posteriorly (Fig. 12d). Mesocoxae closed laterally to mesepimeron. Mesosternum not produced vertically, without anterior tubercle or sulcus; with pronounced lateral projections into mesocoxae. Apex of terminal ventrite subtruncate without notch.

#### Remarks.

This species is very distinctive among the Fijian *Ceresium* by having very long antennae (extending beyond the elytral apices by more than 5 segments), by the laterally multi-tuberculate pronotum, and by the post-medial black macula on each elytron. It is most similar to *Ceresium tuberculatum* in the key characters but can be distinguished from that species by having the pronotum strongly arcuate with dense, white pubescence at the sides (the pronotum in *Ceresium tuberculatum* is quadrate with patchy yellow pubescence on sides and posterior margin). This species is endemic to Fiji and known only from Viti Levu where both known specimens were taken at lights in October (Dillon and Dillon 1952).

### Ceresium scutellaris

Taxon classificationAnimaliaColeopteraCerambycidae

Dillon & Dillon, 1952

Fig. 13

Ceresium scutellaris : Dillon and Dillon 1952: 19, Fiji: Viti Levu, Nandarivatu, holotype (BPBM).

#### Description.

**Figure 13.**
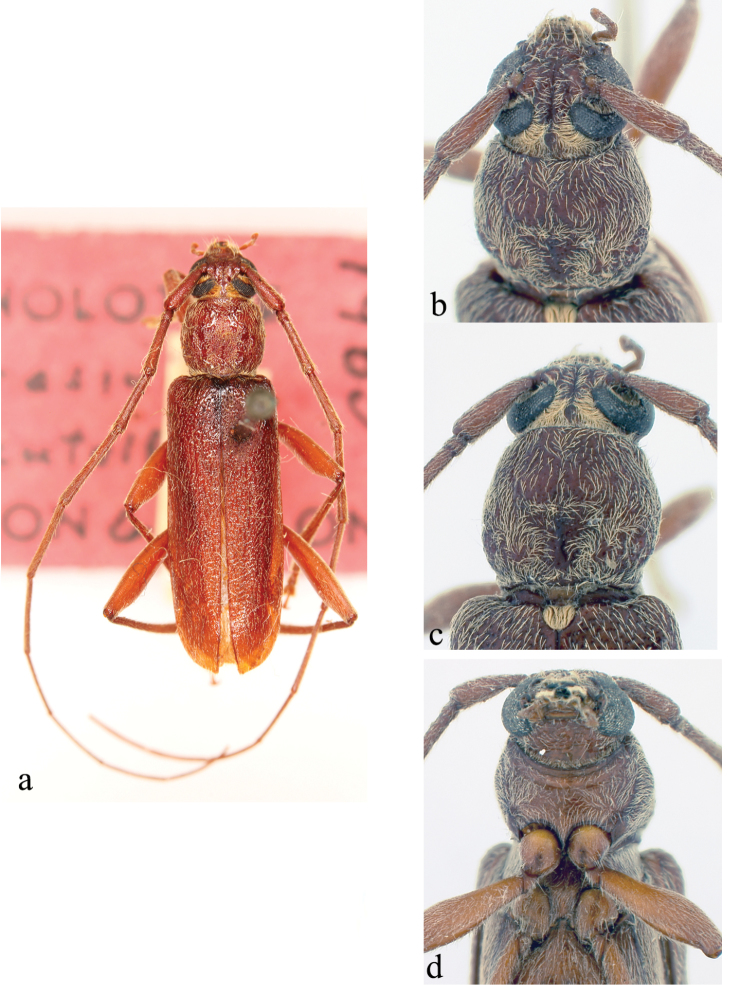
*Ceresium scutellaris* Dillon & Dillon: **a** dorsal habitus (holotype, BPBM) **b** head detail **c** pronotal detail **d** ventral detail.

Based on the holotype specimen (BPBM) and four specimens from 1981 and 2004 surveys (FNIC, USP). *Size* 11.5–16.5 mm long, 2.2–3.5 mm wide at humeri; integument color light brown (Fig. 13a). *Head* with very shallow interantennal tubercle region, tubercles only slightly raised; punctate with very sparse golden pubescence on tubercles and throughout frons; vertex and occiput with dense golden pubescence and a glabrous median line running longitudinally. Golden pubescence denser around eye margins. Frons and frontoclypeal margin densely, coarsely punctate with sparse, long, golden hairs (Fig. 13b). *Antennae* long, extending beyond elytra by 3–4 antennomeres. Antennae with vestiture of short, dense, ochraceous setae (longer at apices of antennomeres). Antennomeres unspined and expanded at apices except for antennae 9–11; last antennomere about 1.2 times length of penultimate. Antennomere 3 shorter than scape; 5 very long and the longest. Scape long, clavate, extending to apical sixth of pronotum.

*Pronotum* arcuate, slightly transverse, apex slightly narrower than base; tubercles absent. No calli. Pronotum with fine, sparse, evenly spaced golden pubescence throughout. Pronotum with fine and dense punctures (Fig. 13c). *Elytron* finely granulate punctures becoming shallower and indistinct towards apex. Sparse golden pubescence, evenly spaced throughout. Elytral apex rounded to suture. Scutellum parallel-sided and then broadly rounded at apex, covered with dense, golden pubescence. *Legs* moderate in length, femora distinctly but gradually clavate, hind femora extending to beyond base of fourth ventrite.

*Venter* of abdomen and thorax with moderately dense, ochraceous pubescence at sides, but sparse pubescence along middle, except for prosternum which is sparsely pubescent throughout. Prosternal process very, weakly notched and not expanded at apex. Procoxal cavities open posteriorly (Fig. 13d). Mesocoxae closed laterally to mesepimeron. Mesosternum declivous, without anterior tubercle, and sulcate anteriorly. Apex of terminal ventrite truncate to unevenly rounded, without notch.

#### Remarks.

This species was not included in the key in the original publication (Dillon and Dillon 1952). It is distinct, along with *Ceresium olidum*, in having an incomplete prosternal process between the procoxae. It is distinguished from that species by having the pronotum with uneven punctation, the third antennal segment extending nearly to the posterior margin of pronotum, and having the head, pronotum and scutellum with dense yellowish tomentum. In *Ceresium olidum*, the pronotum has uniform, dense punctation, the third antennal segment extends to about the midpoint of pronotum, and the head, pronotum and scutellum has fine, sparse, ochraceous pubescence. This species is endemic to Fiji and known only from Viti Levu where specimens have been taken on dead branches and at lights (Dillon and Dillon 1952).

### Ceresium striatipenne

Taxon classificationAnimaliaColeopteraCerambycidae

Dillon & Dillon, 1952

Fig. 14

Ceresium striatipenne : Dillon and Dillon 1952: 15, Fiji: Viti Levu, Nandarivatu, holotype (BPBM).

#### Description.

**Figure 14.**
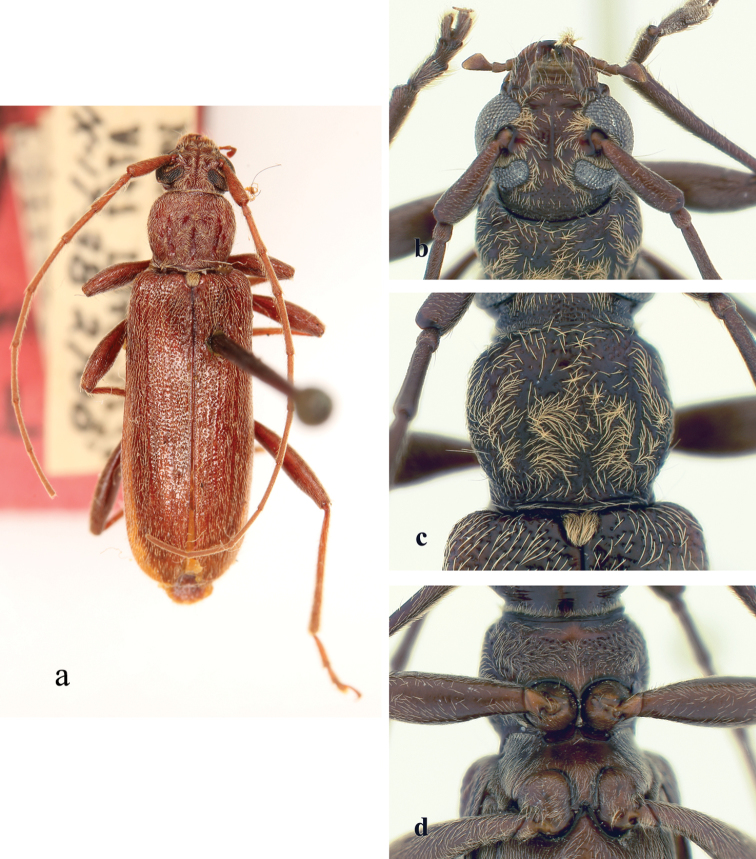
*Ceresium striatipenne* Dillon & Dillon: **a** dorsal habitus (holotype, BPBM) **b** head detail **c** pronotal detail **d** ventral detail.

Based on the holotype and a paratype specimen (BPBM) and two specimens from 2008 surveys (FNIC, USP). *Size* 9.5–11.0 mm long, 2.0–2.5 mm wide at humeri; integument color maroon-brown; darker at head becoming paler towards elytral apex (Fig. 14a). *Head* with shallow interantennal tubercle region, tubercles only slightly raised; punctate with very sparse golden pubescence on tubercle margin; vertex and occiput with sparser almost bare golden pubescence and punctate. Distinct median line running longitudinally between eye lobes. Golden pubescence denser around eye margins (Fig. 14b). *Antennae* long, extending beyond elytra by one antennomere. Antennae with vestiture of short, dense, ochraceous setae (longer at apices of antennomeres). Antennomeres unspined and expanded at apices; last antennomere slightly shorter than penultimate. Antennomere 3 shorter than scape; 4 subequal in length to scape; 5 very long and the longest. Scape long, clavate, extending to apical sixth of pronotum.

*Pronotum* broadly arcuate, widest below middle, and transverse. Narrower at anteriorly than posteriorly. Pronotum with three longitudinally glabrous lines with moderately dense golden pubescence between glabrous lines and sparsely pubescent elsewhere. Pronotum with moderately dense punctures in center between glabrous lines and sparsely elsewhere (Fig. 14c). *Elytron* glabrous with sparse golden pubescence. Disk coarsely, irregularly punctate, punctures finer behind apical quarter. Elytral apex together rounded. Scutellum broadly rounded, covered with dense, golden pubescence. *Legs* small to moderate in length, femora distinctly but gradually clavate, hind femora extending to beyond base of fourth ventrite.

*Venter* of abdomen and thorax with sparse ochraceous pubescence at sides with sparser pubescence along middle. Prosternal process narrow, weakly notched and expanded at apex. Procoxal cavities closed posteriorly (Fig. 14d). Mesocoxae closed laterally to mesepimeron. Mesosternal process broad, slightly expanded at apex and inserted into mesocoxa. Mesosternum gradually declivous, without anterior tubercle, and sulcate anteriorly. Apex of terminal ventrite truncate to unevenly rounded, without notch.

#### Remarks.

The distinctive, longitudinal, glabrous striae characterize this species. The only other species with semi-regular glabrous areas on the elytra is *Ceresium vacillans*, but in that species these regions are in the form of spots rather than lines. This species is endemic to Fiji and known only from Viti Levu where the type specimens were collected at lights in August (Dillon and Dillon 1952).

### Ceresium thyra

Taxon classificationAnimaliaColeopteraCerambycidae

Dillon & Dillon, 1952

Fig. 15

Ceresium thyra : Dillon and Dillon 1952: 21, Fiji: Viti Levu, Tailevu, holotype (BPBM).

#### Description.

**Figure 15.**
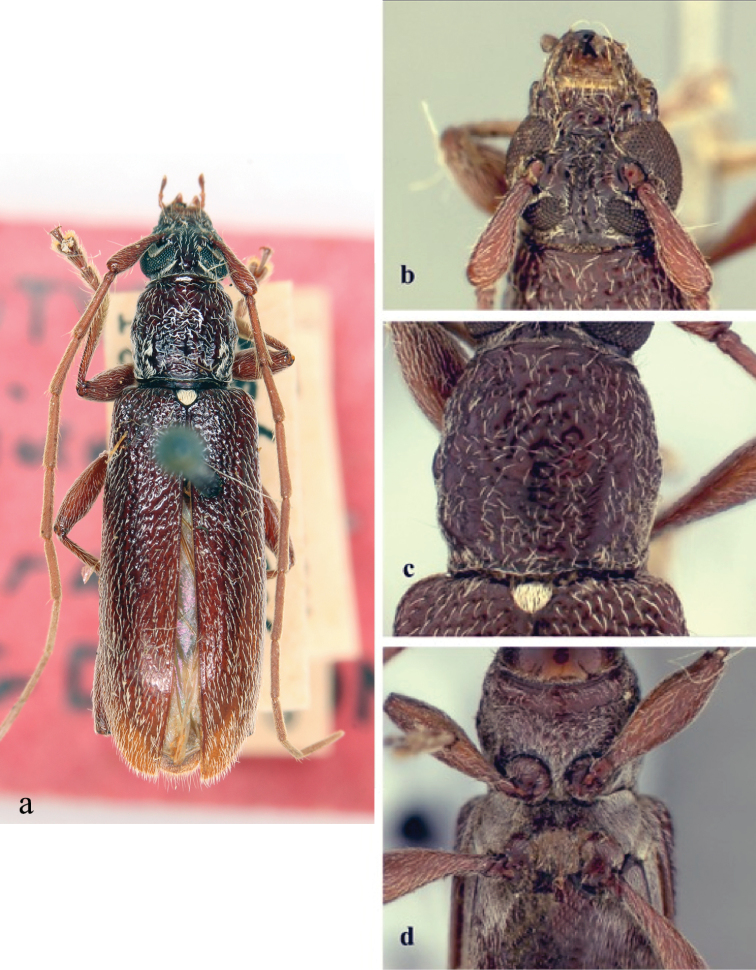
*Ceresium thyra* Dillon & Dillon: **a** dorsal habitus (holotype, BPBM) **b** head detail **c** pronotal detail **d** ventral detail.

Based on photograph of the holotype specimen (BPBM) and five specimens from 1992 and 2004–2005 surveys (FNIC, USP). *Size* 13.0 mm long, 3.0 mm wide at humeri; integument color maroonish brown (Fig. 15a). *Head* with shallow interantennal tubercle region, tubercles only slightly raised; coarsely punctate with very sparse ochraceous pubescence on tubercles and throughout frons; vertex and occiput with sparser ochraceous pubescence almost bare. Ochraceous pubescence denser around eye margins. Frons and frontoclypeal margin densely, coarsely punctate with sparse, long, ochraceous hairs (Fig. 15b). *Antennae* long, extending beyond elytra by 2 antennomeres. Antennae with vestiture of short, dense, ochraceous setae (longer at apices of antennomeres). Antennomeres unspined and not expanded at apices; last antennomere about 1.1 times length of penultimate. Antennomere 4 shorter than scape; 3 almost subequal in length to scape; 5 longest. Scape long, clavate, extending to apical quarter of pronotum.

*Pronotum* arcuate, widest across middle, and almost as long as wide. No tubercles or callus present. Pronotum with sparse ochraceous pubescence. Pronotum with sparse, ill-defined punctures (Fig. 15c). *Elytron* with sparse, evenly spaced ochraceous pubescence. Punctation shallow, sparse, gradually becoming shallower and indistinct towards apex. Elytral apex narrowly rounded to suture. Scutellum narrowly rounded, covered with dense, white pubescence. *Legs* moderate in length, femora distinctly but gradually clavate, hind femora extending to beyond base of fourth ventrite.

*Venter* of abdomen with sparse white pubescence and metasternum with sparse white pubescence at center and moderately dense on sides. Mesosternum also moderately dense with white pubescence. Prosternal process moderately narrow, vertical and acutely declivous, approximately 1/3 width of procoxa, notched and expanded at apex. Procoxal cavities open posteriorly (Fig. 15d). Mesocoxae closed laterally to mesepimeron. Mesosternum process broad, declivous but not tuberculate and sulcate anteriorly; at apex each side expands into a triangular tooth inserted into mesocoxae. Metasternum with black line running full length longitudinally along middle. Apex of terminal ventrite truncate with a small bump in middle.

#### Remarks.

The very dense, nearly white pubescence of the scutellum is distinctive, along with the dorsally callous pronotum. This species is endemic to Fiji and known only from Viti Levu where specimens were collected in September (Dillon and Dillon 1952).

### Ceresium tuberculatum

Taxon classificationAnimaliaColeopteraCerambycidae

Waqa & Lingafelter, 2009

Fig. 16

Ceresium tuberculatum : Waqa and Lingafelter 2009: 4, Fiji: Gau, holotype (BPBM).

#### Description.

**Figure 16.**
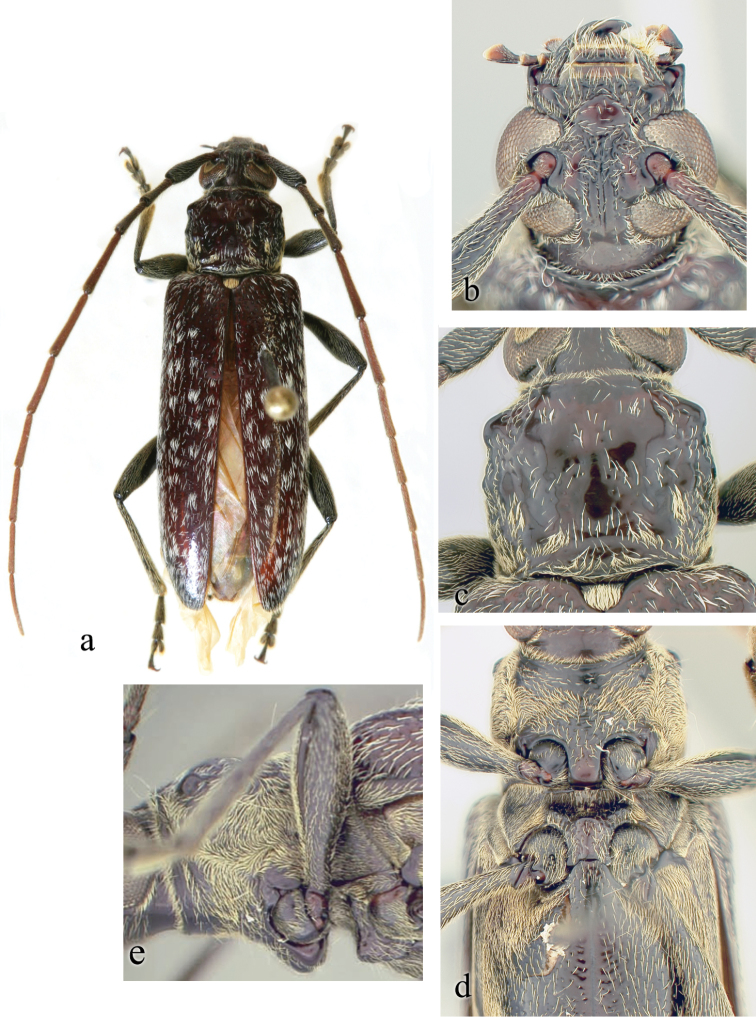
*Ceresium tuberculatum* Waqa & Lingafelter: **a** dorsal habitus, male paratype **b** head detail, male **c** pronotal detail, male **d** ventral view showing width of prosternal and mesosternal intercoxal processes, along with pubescence distribution **e** lateral view showing acutely declivous prosternal process, weakly tuberculate, acutely declivous mesosternal process, and laterally closed mesocoxa.

Based on the holotype specimen (BPBM), 15 paratypes from 2005 surveys (FNIC, USP), and original description of Waqa and Lingafelter (2009). *Size* 14.0–18.0 mm long, 3.5–4.5 mm wide at humeri; integument color dark reddish brown (occasionally piceous) (Fig. 16a). *Head* with shallow interantennal tubercle region, tubercles only slightly raised; punctate with very sparse ochraceous pubescence on tubercles and throughout frons; vertex and occiput with sparser ochraceous pubescence. Ochraceous pubescence denser around eye margins. Frons and frontoclypeal margin densely, coarsely punctate with sparse, long, ochraceous hairs. *Antennae* long, extending beyond elytra by 3–4 antennomeres (longer in males than females). Antennae with vestiture of short, dense, ochraceous setae (longer at apices of antennomeres). Antennomeres unspined and not expanded at apices; last antennomere approximately 1.4 times length of penultimate in males (about 1.2 times length of penultimate in females). Antennomere 3 and 4 each shorter than scape; 5–9 longest except for 11 and subequal in length. Scape long, clavate, extending to apical fifth of pronotum.

*Pronotum* quadrate, slightly widest anteriorly, and slightly wider than long; tuberculate at sides; constricted subbasally and apically. Raised tubercles present at middle of sides and anterolaterally. Three poorly-defined calli on disk: 1 medial and 2 anteromedial between middle callus and anterolateral tubercle. Pronotum with patchy ochraceous pubescence, denser at sides and posterior margin, slightly less dense anteriorly; center of disk mostly glabrous. Pronotum with sparse, poorly-defined punctures in males (except on smooth calli), only sparse depressions present in females (Fig. 16b, c). *Elytron* glabrous except for scattered sparse patches of white (occasionally ochraceous) pubescence. Punctation shallow, sparse, gradually becoming shallower and indistinct towards apex. Elytral apex rounded to suture. Scutellum broadly rounded, covered with dense, ochraceous pubescence. *Legs* moderate in length, femora distinctly but gradually clavate, hind femora extending to beyond base of fourth ventrite.

*Venter* of abdomen and thorax with moderately dense, ochraceous pubescence at sides, but mostly glabrous along middle, except for prosternum which is densely pubescent. Prosternal process broad, vertical and acutely declivous, approximately 1/3 width of procoxa, weakly notched and expanded at apex. Procoxal cavities open posteriorly. Mesocoxae closed laterally to mesepimeron. Mesosternum rather acutely declivous, with small anterior tubercle, and sulcate anteriorly (Fig. 16d, e). Apex of terminal ventrite in males with median notch; in females truncate to unevenly rounded, without notch.

#### Remarks.

The prominent anterolateral pronotal tubercles, narrowly tapering pronotum posteriorly, and acutely declivous prosternal process are distinctive for this species. This recently described species is endemic to Fiji and known only from Gau and Viti Levu Islands where specimens have been collected in Malaise traps, mostly, from April through June and October through November (Waqa and Lingafelter 2009).

### Ceresium unicolor

Taxon classificationAnimaliaColeopteraCerambycidae

(Fabricius, 1787)

Fig. 17

Saperda unicolor : Fabricius 1787: 147, Amsterdam Island, French Southern Islands, holotype (BMNH).

#### Description.

**Figure 17.**
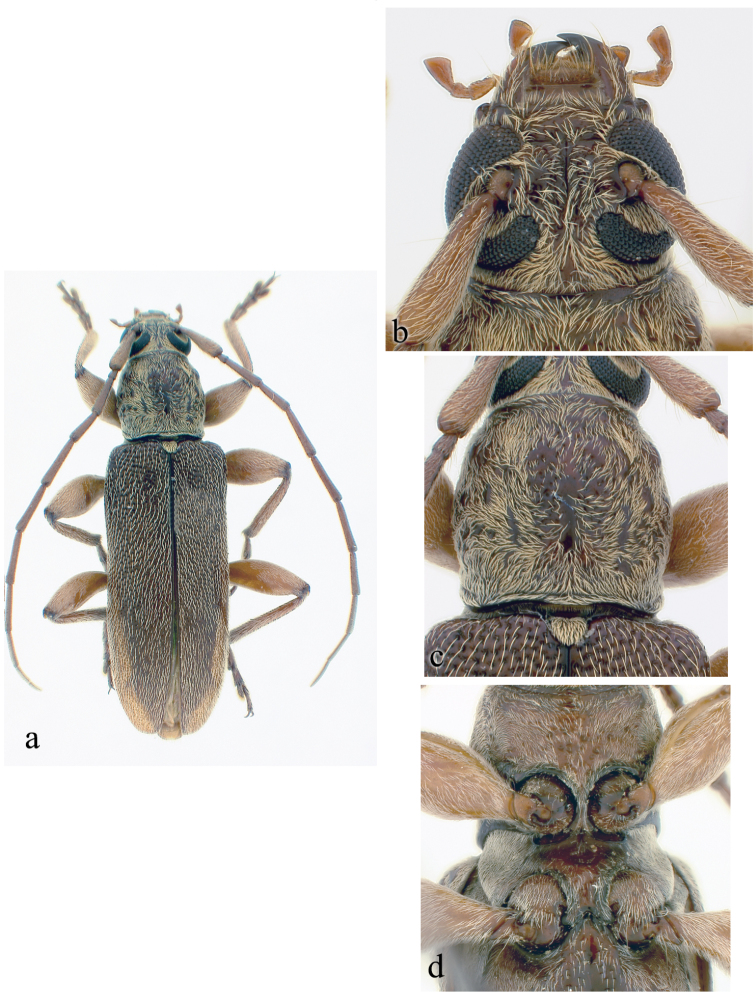
*Ceresium unicolor* (Fabricius): **a** dorsal habitus **b** head detail **c** pronotal detail **d** ventral detail.

Based on the original description (Fabricius 1787) and specimens from 1938, 2005 and 2007 surveys (FNIC, USP). *Size* 15.0–17.0 mm long, 3.0–4.0 mm wide at humeri; integument color orangish-brown to maroon-brown (occasionally piceous) (Fig. 17a). *Head* with shallow interantennal tubercle region, tubercles only slightly raised; punctate with moderately dense yellow pubescence on tubercles; vertex and occiput with sparser yellow pubescence. Yellow pubescence denser around eye margins. Frons and frontoclypeal margin densely, coarsely punctate with sparse, long, yellow hairs (Fig. 17b). *Antennae* long, extending beyond elytra by 1–2 antennomeres. Antennae with vestiture of short, dense, ochraceous setae (longer at apices of antennomeres). Antennomeres unspined and slightly expanded at apices; last antennomere subequal in length of penultimate. Antennomere 3 and 4 each shorter than scape; 3 shortest; 5–9 longest and subequal in length. Scape long, clavate, extending to apical sixth of pronotum.

*Pronotum* broadly arcuate quadrate, widest across middle, and as long as wide. Tubercles absent. Pronotum with a broad median line and a fine line slightly half-way between middle and sides on each side of pronotum. Pronotum with moderately dense to dense yellow pubescence. Pronotum with sparse, coarse punctures (Fig. 17c). *Elytron* with sparse and evenly spaced yellow pubescence throughout. Punctation shallow, sparse, gradually becoming shallower and smaller in size towards apex. Elytral apex rounded to suture. Scutellum broadly rounded, covered with dense, yellow pubescence. *Legs* moderate in length, femora distinctly but gradually clavate, hind femora reaching base of fourth ventrite.

*Venter* of abdomen and metasternum with sparse yellow pubescence towards center and becoming denser on sides. Prosternal process moderately narrow, vertical and acutely declivous, about 1/4 width of procoxa, weakly notched and not expanded at apex. Procoxal cavities open posteriorly (Fig. 17d). Mesocoxae closed laterally to mesepimeron. Mesosternum rather acutely declivous, with small anterior tubercle, and sulcate anteriorly. Mesosternal process expanded at base into tubular tooth inserted into mesocoxa. Apex of terminal ventrite truncate with a slight bump towards middle.

#### Remarks.

This species is somewhat variable and lacking a suite of very distinctive characters. In the key, it is distinguished by the pronotum lacking maculae and lateral tubercles but having several dorsal calli, and having pubescence of the head, pronotum, elytra and scutellum similar in density and coloration. This species is widespread and known from Mauritius, Seychelles, New Zealand, Waigeo Island, Papua New Guinea, Bismarck Archipelago, Solomon Islands, Vanuatu, Hawaii, and Fiji (Bigger and Schofield 1983). In Fiji, it is known known from Viti Levu, Taveuni, Lau Islands, and Vanua Levu where specimens have been collected throughout the year, most commonly at lights (Dillon and Dillon 1952).

### Ceresium vacillans

Taxon classificationAnimaliaColeopteraCerambycidae

Dillon & Dillon, 1952

Fig. 18

Ceresium vacillans : Dillon and Dillon 1952: 24, Fiji: Lau Islands, Thikombia, holotype (BPBM).

#### Description.

**Figure 18.**
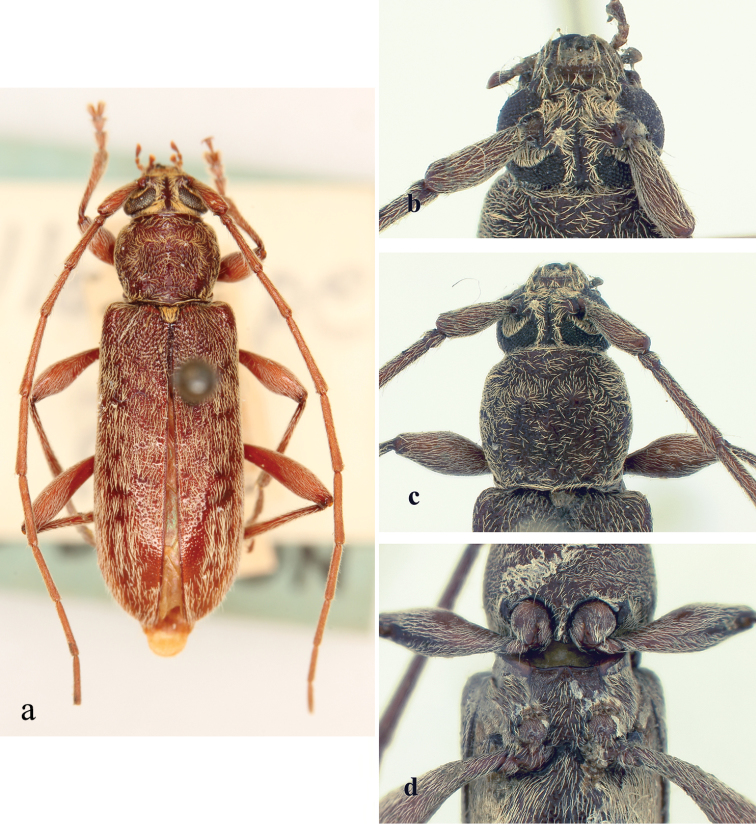
*Ceresium vacillans* Dillon & Dillon: **a** dorsal habitus (holotype, BPBM) **b** head detail **c** pronotal detail **d** ventral detail.

Based on the holotype specimen (BPBM) and a specimen from 1998 survey (USP). *Size* 9.0–13.0 mm long, 2.0–3.0 mm wide at humeri; integument color maroonish brown (Fig. 18a). *Head* with slightly deep interantennal tubercle region, tubercles only slightly raised; punctate with moderately dense yellow pubescence on tubercles and throughout frons; vertex and occiput bare. Distinct median line between eye lobes. Yellow pubescence denser around eye margins and basal head margin. Frons and frontoclypeal margin moderately dense and coarsely punctate with sparse, long, yellow hairs (Fig. 18b). *Antennae* long, extending beyond elytra by four antennomeres. Antennae with vestiture of short, dense, ochraceous setae. Antennomeres unspined and expanded at apices; last antennomere just slightly longer than penultimate. Scape shorter than all antennomeres; 5–7 very long and the longest and subequal in length. Scape short, clavate, extending to apical fifth of pronotum.

*Pronotum* arcuate, wider across middle, transverse, apical margin narrower than base. Disk moderately dense with ochraceous pubescence, and coarsely punctate. Medially with a broad transversely ovate impression limited by two low tubercles in front, two behind and a fifth one in its center (Fig. 18c). *Elytron* with irregularly disposed ochraceous pubescence with irregular, small, glabrous maculae. Punctation shallow, dense, gradually becoming shallower and indistinct towards apex. Elytral apex together rounded. Scutellum broadly rounded, covered with dense, ochraceous pubescence. *Legs* moderate in length, femora distinctly but gradually clavate, hind femora just reaching elytral apex.

*Venter* of abdomen with moderately dense, ochraceous pubescence at sides, sparse ochraceous pubescence along middle, except for prosternum which is sparsely pubescent throughout and on sides. Prosternal process broad, vertical and acutely declivous, about 1/4 width of procoxa, weakly notched and expanded at apex. Procoxal cavities open posteriorly (Fig. 18d). Mesocoxae closed laterally to mesepimeron. Mesosternal process broad, expanded at apex, inserted into mesocoxa. Mesosternum rather acutely declivous, with small anterior tubercle, and sulcate anteriorly. Apex of terminal ventrite truncate to unevenly rounded, without notch.

#### Remarks.

This species is most similar to *Ceresium striatipenne* in that it possesses glabrous regions on the elytra, however in this species, the glabrous regions are irregular and small rather than forming narrow lines as in *Ceresium striatipenne*. This species is endemic to Fiji and known only from Viti Levu and the Lau Islands where specimens have been collected in July and September (Dillon and Dillon 1952).

### Key to species of *Ceresium* of Fiji

Diagnostic features in the key include easily coded character states: presence/absence of a macula on the pronotum; shape and color of the macula on pronotum; shape of pronotum (i.e. tuberculate, strongly/weakly arcuate); color of pubescence on pronotum; density of pubescence on pronotum; pronotum with or without calli; and elytra with/without a glabrous line or macula.

**Table:** 

1	Pronotum with yellow macula	**2**
–	Pronotum without yellow macula	**3**
2(1)	Pronotum with two dense areas of yellowish pubescence on either side, subequal in length	***Ceresium guttaticolle* (Fairmaire)**
–	Pronotum with three or four areas of yellowish pubescence on either side, apical one largest	***Ceresium nigroapicale* Dillon & Dillon**
3(1)	Pronotum tuberculate laterally	**4**
–	Pronotum not tuberculate	**5**
4(3)	Pronotum strongly arcuate with white pubescence denser on either lateral sides	***Ceresium repandum* Dillon & Dillon**
–	Pronotum quadrate, posteriorly narrowed, with patchy yellow pubescence denser on either sides and posterior margin	***Ceresium tuberculatum* Waqa & Lingafelter**
5(3)	Pronotum with calli	**6**
–	Pronotum without calli	**7**
6(5)	Head and pronotum with moderately dense yellowish pubescence. Scutellum with pubescence of similar color as that on pronotum and elytra	***Ceresium unicolor* (Fabricius)**
–	Head and pronotum with sparse ochraceous pubescence. Scutellum with much paler pubescence than on pronotum and elytra	***Ceresium thyra* Dillon & Dillon**
7(5)	Mesosternal process tuberculate anteriorly	**8**
–	Mesosternal process not tuberculate	**9**
8(7)	Pronotum quadrate in shape. Integument rather opaque, not strongly shining. Large species (>21 mm in length)	***Ceresium grandipenne* Fairmaire**
–	Pronotum uniformly rounded laterally. Integument very shiny. Moderate to small species (<20 mm in length)	***Ceresium pubescens* Dillon & Dillon**
9(7)	Elytra with glabrous lines or spots between pubescence	**10**
–	Elytra without glabrous lines or maculae	**11**
10(9)	Elytra with fine glabrous linear regions between pubescence. Prosternal process very narrow between procoxae	***Ceresium striatipenne* Dillon & Dillon**
–	Elytra with small scattered glabrous spots between pubescent patches. Prosternal process moderately wide between procoxae	***Ceresium vacillans* Dillon & Dillon**
11(9)	Prosternal process incomplete between procoxae	**12**
–	Prosternal process fully extending between procoxae	**13**
12(11)	Pronotum with uneven punctation. Third antennal segment extending nearly to posterior margin of pronotum. Head, pronotum and scutellum with dense yellowish tomentum	***Ceresium scutellaris* Dillon & Dillon**
–	Pronotum with uniform, dense punctation. Third antennal segment extending to about midpoint of pronotum. Head, pronotum and scutellum with finely sparse ochraceous pubescence	***Ceresium olidum* (Fairmaire)**
13(11)	Pronotum strongly arcuate laterally	**14**
–	Pronotum weakly arcuate/feebly elongate	**15**
14(13)	Pronotum almost hexagonal in shape, with a diffuse, dark macula in the integument either side of middle, widest before middle	***Ceresium lucidum* Dillon & Dillon**
–	Pronotum with sides broadly rounded, without maculae on integument, widest medially	***Ceresium epilais* Dillon & Dillon**
15(13)	Elytra with apical third paler than rest	***Ceresium gracilipes* Fairmaire**
–	Elytra uniformly colored	**16**
16(15)	Mesosternal process basal notch parallel sided. Pronotum with narrow, glabrous, impunctate line at middle restricted to posterior half	***Ceresium promissum* Dillon & Dillon**
–	Mesosternal process basal notch at an angle. Pronotum with narrow, glabrous, impunctate line at middle centrally located	***Ceresium decorum* Dillon & Dillon**

## Supplementary Material

XML Treatment for Ceresium decorum

XML Treatment for Ceresium epilais

XML Treatment for Ceresium gracilipes

XML Treatment for Ceresium grandipenne

XML Treatment for Ceresium guttaticolle

XML Treatment for Ceresium lucidum

XML Treatment for Ceresium nigroapicale

XML Treatment for Ceresium olidum

XML Treatment for Ceresium promissum

XML Treatment for Ceresium pubescens

XML Treatment for Ceresium repandum

XML Treatment for Ceresium scutellaris

XML Treatment for Ceresium striatipenne

XML Treatment for Ceresium thyra

XML Treatment for Ceresium tuberculatum

XML Treatment for Ceresium unicolor

XML Treatment for Ceresium vacillans
